# Complex housing causes a robust increase in dendritic complexity and spine density of medial prefrontal cortical neurons

**DOI:** 10.1038/s41598-018-25399-4

**Published:** 2018-05-09

**Authors:** Archana Ashokan, Jamien Wee Han Lim, Nicholas Hang, Rupshi Mitra

**Affiliations:** School of Biological Sciences, Nanyang Technological University, 60 Nanyang Drive, Singapore, 637551 Singapore

## Abstract

Prelimbic cortex and infralimbic cortex, parts of the ventromedial prefrontal cortex, are critical brain regions for generating a flexible behavioral response to changing environmental contingencies. This includes the role of these brain structures in the extinction of learned fear, decision making and retrieval of remote memories. Dendritic structure of medial prefrontal cortex neurons retains significant structural plasticity in adulthood. This has been mainly demonstrated as dendritic atrophy and loss of dendritic spines due to chronic stress. It remains unknown if housing condition of the animals itself can cause opposing changes in the dendritic organization. In that backdrop, here we report that short-term increase in complexity of the housing causes a robust increase in complexity of dendritic architecture of prelimbic and infralimbic neurons. This is reflected in the dendritic expansion of prelimbic neurons and increase in spine density of prelimbic and infralimbic neurons. These results suggest that non-invasive changes in the housing environment can be harnessed to study brain reserves for the flexible and species-typical behaviors.

## Introduction

Medial prefrontal cortex is a critical brain structure for mediation of flexible behaviors. This includes its role in decision making and retrieval of remote memories^1^. Similarly, medial prefrontal cortex provides behavioral inhibition during extinction of conditioned fear^2^. Lesions of medial prefrontal cortex in rodents produce deficits in species-typical behaviors like hoarding of food^3^ and nest building^4^.

Neurons in the medial prefrontal cortex exhibit remarkable capacity of structural plasticity in response to aversive behavioral experiences. For example, chronic restraint stress results in approximately 20% reduction in total dendritic length of apical dendrites in medial prefrontal cortex neurons^5^. This atrophy co-occurs with loss of spines by ≈16% relative to unstressed controls^6^. These numerical estimates suggest that chronic restraint can result in loss of around one-third of all spines in the medial prefrontal cortex; representing a significant retraction^6^. This is corroborated by reduction of glutamatergic synaptic transmission in prefrontal cortex neurons by chronic stress and compromise in temporal order recognition memory^7^. Similarly, exogenous corticosterone treatment causes atrophy and spine loss, although in this case proximal dendrites are spared, and distal dendrites show more consistent effects^8,9^. These studies suggest that prefrontal neurons in adulthood retain a capacity to undergo structural changes in response to the incipient stressful environment.

It remains hitherto unknown if the sensory and social complexity of the living environment itself can change the dendritic organization of prefrontal cortex. In this report, we experimentally test this possibility by determining the effects of complex housing environment on dendritic architecture and spine density of prelimbic and infralimbic regions of the medial prefrontal cortex.

## Materials and Methods

### Animals and Experimental groups

Male Wistar rats were randomly assigned to experimental groups (7 weeks old at the start of experiments). Animals were maintained in a light-dark cycle of 12 h (light on 0700 h) with *ad libitum* food and water. All experimental procedures were reviewed and approved by the Institutional Animal Care and Use Committee (IACUC) of NTU. All experiments were performed in accordance with IACUC guidelines and regulations. Simple housing consisted of two animals living in a standard animal facility cage (37 × 22 × 18 cm). Complex environment consisted of larger cages (72 × 51 × 110 cm), more animals per cage (4 animals per cage) and presence of novel objects. The novel objects included climbing walls made of wire-net, plastic tunnels, plastic and wooden objects of varied color and texture, ample nesting material, gustatory variety in the form of fruit loops and sunflower seeds and layered tiers within the cage. Running wheel was not provided in the complex housing. The arrangement of the objects was changed every fourth day. Animals were either transferred from standard housing to complex housing or retained in simple and standard housing for twenty-one successive days. Control animals remained in the same dyad that was established prior to the start of the experiments. For animals exposed to complex housing, two previously established dyads were transferred together to create a cohort of four animals per cage. Length of exposure to the complex housing and frequency of changing the objects was guided by our earlier studies that show the potential of the protocol used here to elicit changes in dendritic parameters^10^. Adult animals were used in the current experiment to restrict analysis away from the peripubertal phase of brain plasticity. Control animals were not handled during the experimentation except routine cage change. Animals in complex housing were not handled except brief episodes of rearrangement of objects every fourth day.

### Golgi-Cox staining for medial prefrontal neurons

Brains were freshly harvested on day 22 and were stained using commercially available Golgi-Cox reagents following the manufacturer’s instructions (FD Neurotechnologies, USA). Stained brains were cryosectioned in coronal planes at the thickness of 100 μm. Sections spanning bregma levels from 3.72 mm to 3.00 mm were used for further analysis of the medial prefrontal cortex neurons. Sections were dehydrated in a graded series of alcohol, cleared in xylene and then cover slipped using a non-aqueous Permount mounting medium (Fisher Scientific, USA) on superfrost glass slides.

### Quantification of dendritic complexity

**Figure 1:**
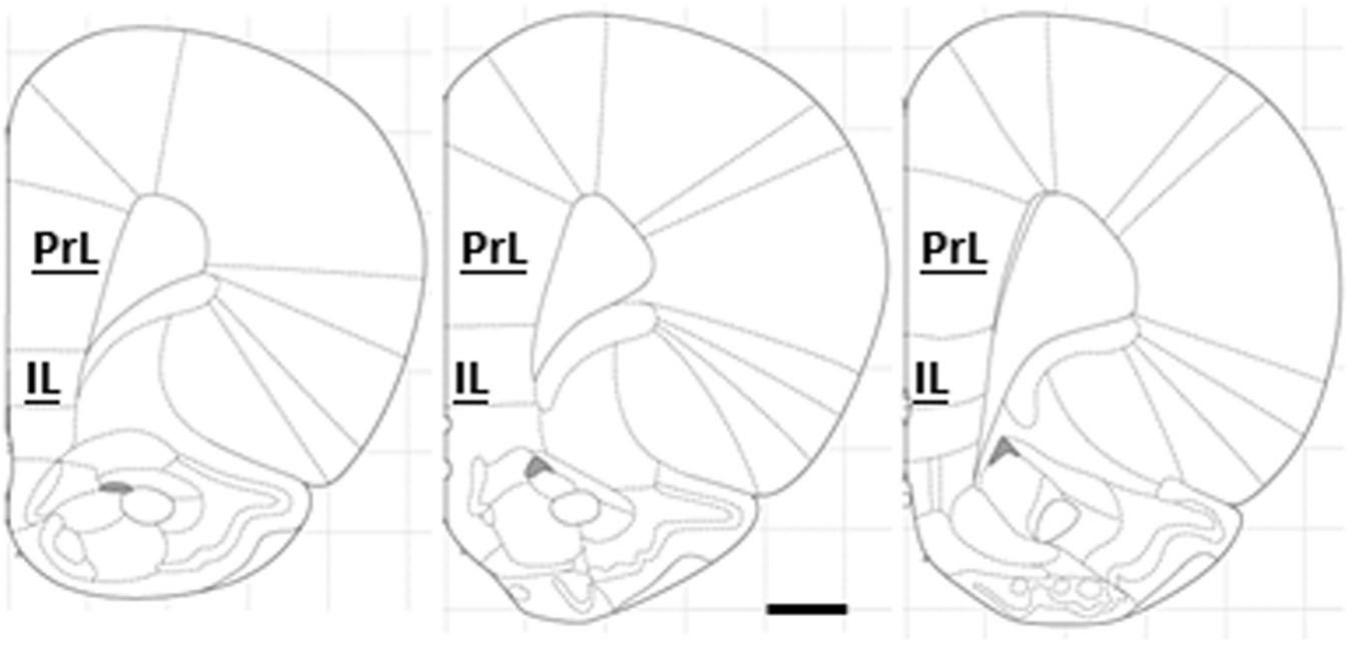
Anatomical bounds of prelimbic (PrL) and infralimbic (IL) cortex used for sampling of the dendritic trees. Only one hemisphere is depicted, at coronal planes of bregma 3.72 mm, 3.24 mm and 3.00 mm (left to right). Outlines adapted from^46^. Scale bar = 1 mm.

Pyramidal neurons from the prelimbic and infralimbic regions of the medial prefrontal cortex were analyzed (PrL and IL, respectively). Figure 1 depicts anatomical boundaries of PrL and IL on representative coronal planes. Mean of six to eight neurons was used to estimate dendritic parameters for each animal.

**Figure 2:**
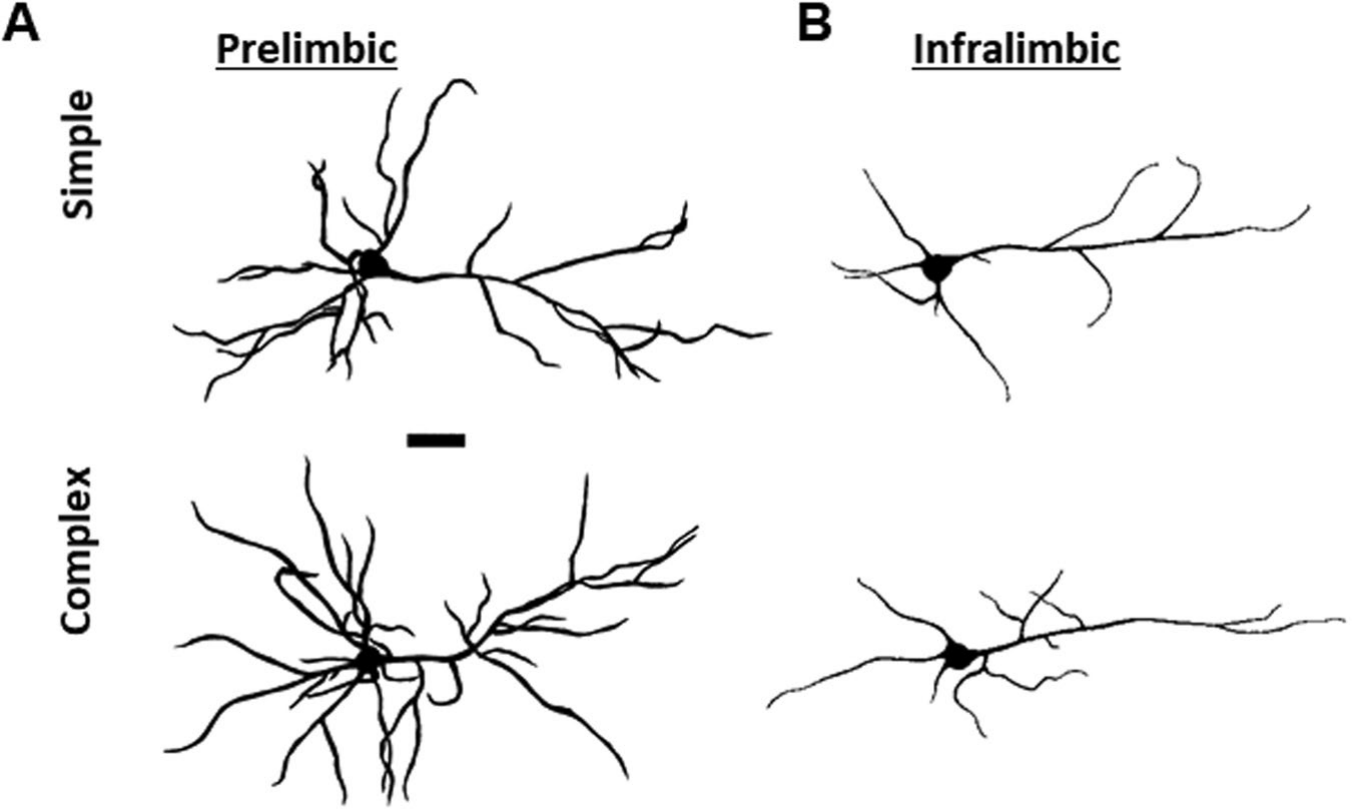
Representative dendritic profiles from animals reared in simple and complex environments. Scale bar = 100 µm.

Six to eight neurons from each animal were randomly chosen for the analysis, excluding neurons with truncated dendrites or uneven impregnation. Two-dimensional profiles of dendritic arbors were drawn at 400X magnification using a camera lucida attachment on the optical microscope (Olympus BX43, Japan). Dendritic profiles were then scanned along with a calibrated scale for subsequent analysis (300 dpi, 8-bit grayscale, tiff) using a freely available image processing package (Fiji, https://fiji.sc/). Figure 2 depicts representative dendritic profiles.

The total dendritic length was quantified by counting foreground pixels in binary skeletonized images. The number of primary dendrites was estimated by counting dendritic intersection with a circular region of interest (radius = 30 µm) centered on the cell soma. A Sholl analysis was further conducted for PrL neurons by overlaying multiple circular regions of interest centered on the cell soma and successively increasing the radius in step size of 10 µm^11^. Number of intersections made by the dendritic arbors was then quantified as a function of radial distance from the soma. A previously described routine in Fiji was used^12^. Total number of intersections, maxima for the number of the intersection and the radius for the maxima of the intersection were recorded.

### Quantification of spine density

Dendritic spines were manually counted at 1000X magnification using an oil-immersion objective lens. Dendrites directly originating from cell soma were classified as primary dendrites, and those arising from primary dendrites were classified as secondary dendrites. Starting from the origin of the branch, and continuing away from cell soma, spines were counted along 80 µm stretch of dendrite for PrL, and 60 µm stretches for IL. Mean of these dendritic segments on different neurons was used to estimate spine density for each animal.

### Data analysis and Statistics

Mean for each animal, across multiple neurons analyzed, was used as biological replicate for statistical analysis. Statistical significance for comparisons between simple and complex housing was calculated using unpaired two-tailed Student’s t-test with *p* < 0.05 considered significant. The standardized effect size was calculated using Cohen’s *d*^13^; with values above the magnitude of one interpreted as being of robust magnitude. Negative *d* values correspond to the comparisons where the mean of complex housing was greater than that of simple housing. Mean inter-group difference was also calculated with 95% confidence intervals. A repeated measure analysis of variance was conducted for Sholl analysis for number of intersections as a function of radial distance from the soma.

Data is graphically presented as mean and standard error of the mean (SEM), along with individual values for each animal for each endpoint. Number of animals in each experimental group is noted in the figure legends.

### Data analysis and Statistics

The datasets generated during and/or analyzed during the current study are available from the corresponding author on reasonable request.

## Results

### Complex environment increased the dendritic complexity of prelimbic medial prefrontal cortex neurons

**Figure 3:**
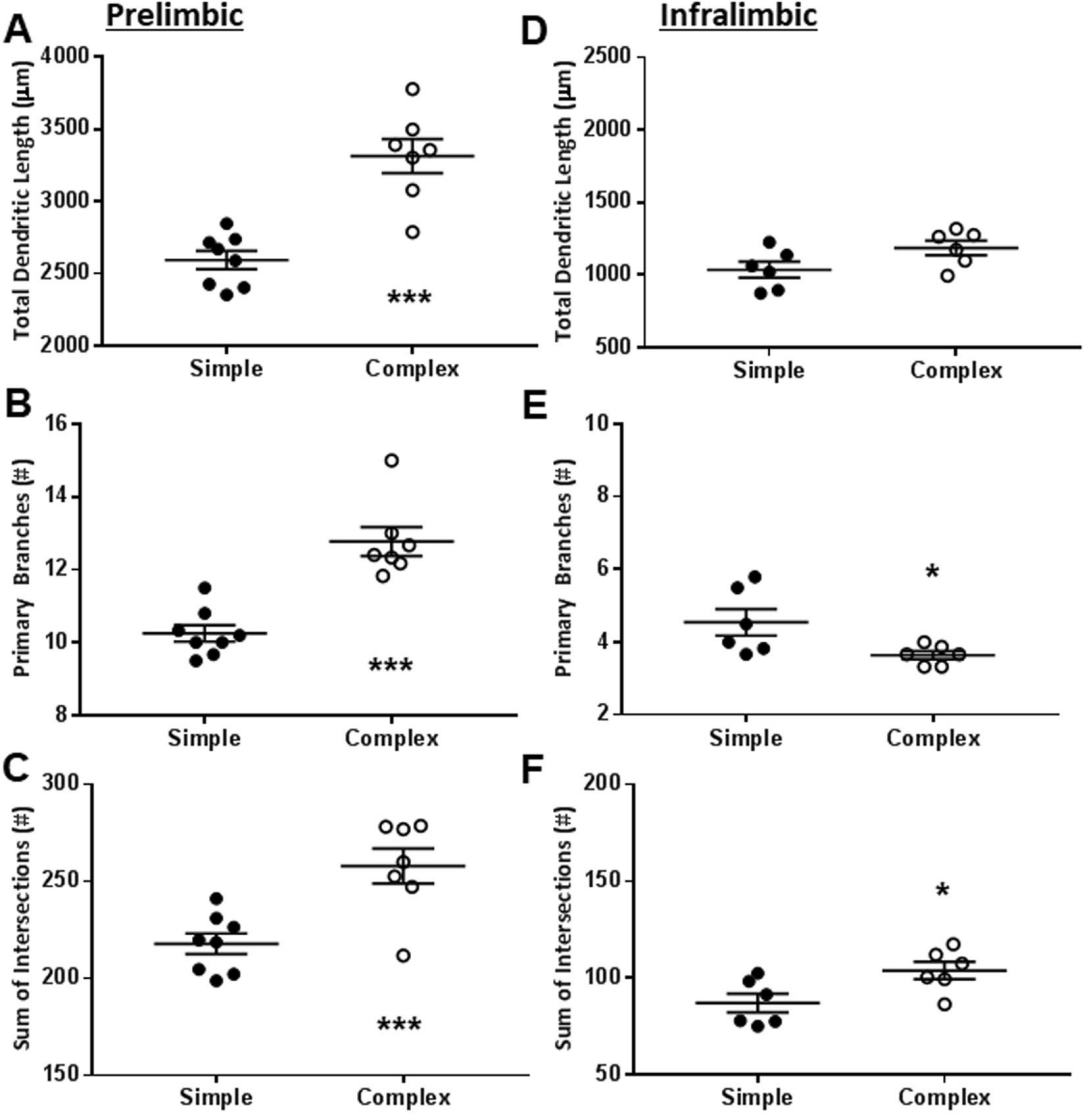
Complex environment increased dendritic complexity of the neurons in prelimbic, but not infralimbic, medial prefrontal cortex. Panels depict mean and SEM, along with individual animals represented as dots. *p < 0.05; and ***p < 0.001; unpaired Student’s t-test. n = 8 animals for simple and 4 for complex environment (prelimbic); and, 6 animals for simple and 6 for complex environment (infralimbic).

Exposure to complex environment significantly increased the dendritic complexity of the pyramidal neurons in prelimbic region of the medial prefrontal cortex (PrL). This was evident by congruent increase in total dendritic length (Fig. 3A; t_13_ = 5.55, *p* < 0.001), number of primary branches (Fig. 3B; t_13_ = 5.70, *p* < 0.001) and total number of intersections with the Sholl grid (Fig. 3C; t_13_ = 3.93, *p* = 0.002). The effects of housing conditions on PrL dendritic complexity were substantial in the magnitude; evidenced by robust effect sizes for total dendritic length (Cohen’s *d* = −2.8; ∆ = 720 µm with 95% confidence intervals 440 to 1001 µm), number of primary branches (Cohen’s *d* = −2.9; ∆ = 2.52 with 95% confidence intervals 1.56 to 3.48) and total number of intersections (Cohen’s *d* = −2.0; ∆ = 40.2 with 95% confidence intervals 18.1 to 62). For all the endpoints, maxima of animals from the simple environment were below the mean for animals from the complex environment (Fig. 3A–C).

Complex housing environment produced equivocal effects on the dendritic complexity of the infralimbic neurons (IL) regarding both probability of type 1 error and directions of the effect size. Effects of housing complexity on the total dendritic length of the IL neurons did not reach statistical significance (Fig. 3D; t_10_ = 2.03, *p* = 0.070). Complex environment reduced the number of primary branches (Fig. 3E; t_10_ = 2.35, *p* = 0.041). In contrast, complex environment significantly increased total number of intersections for IL neurons with the Sholl grid (Fig. 3F; t_10_ = 2.55, *p* = 0.029). The effect size for three endpoints remained robust albeit in opposing directions within individual endpoints (Cohen’s *d* = −1.17 for dendritic length, −1.47 for total number of intersections and 1.35 for number of primary branches).

**Figure 4:**
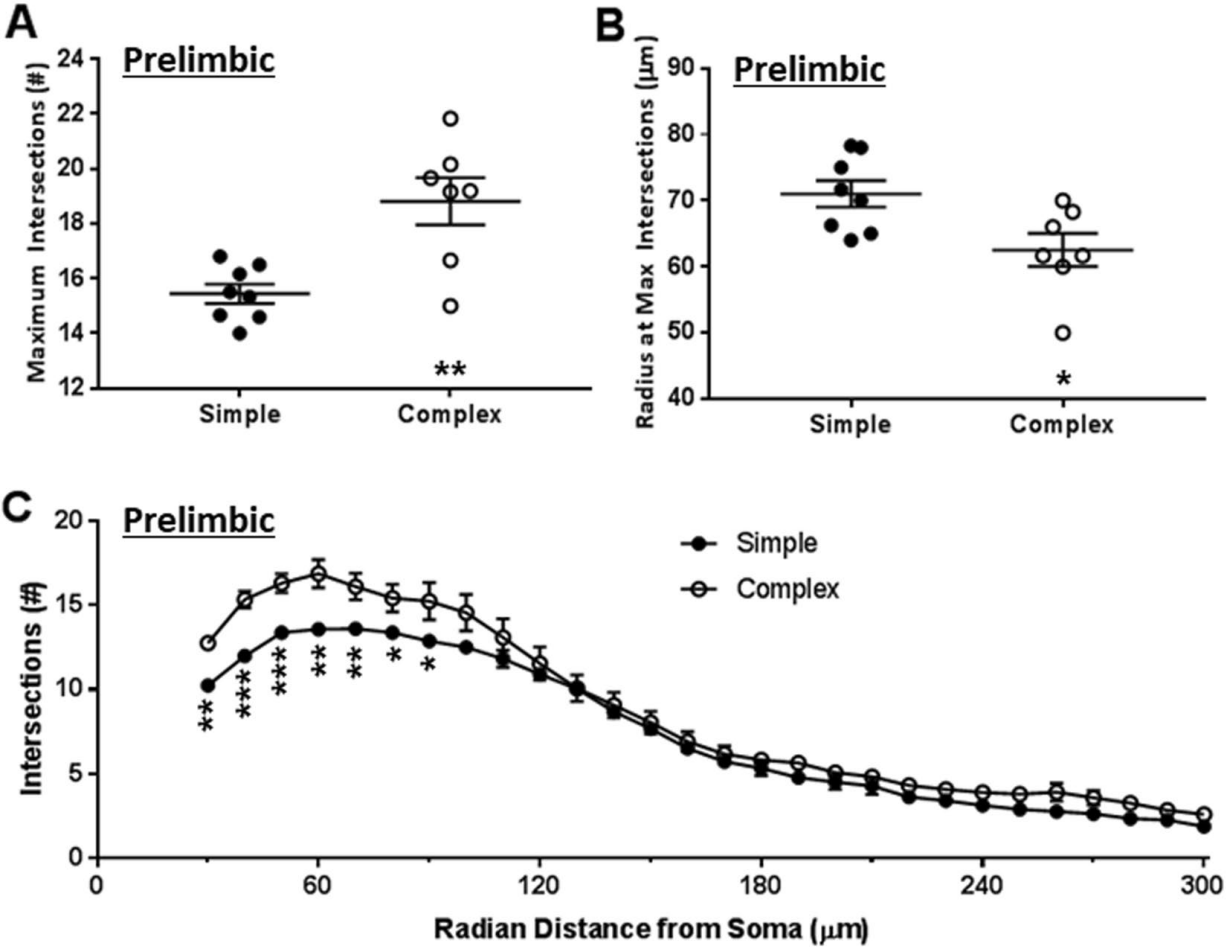
Complex environment caused dendritic expansion proximal to the cell soma. A grid of concentric circles was placed on neurons centered on cell soma (incremental radius = 10 µm). Intersections with successive circles in the grid were counted. Panel A depicts maxima for the number of the intersections. Panel B depicts radius of the circle where maximum radius was encountered. Panel C depicts number of intersections (ordinate) as a function of radial distance from soma (abscissa). **p < 0.05; **p < 0.01; and ***p < 0.001; unpaired Student’s t-test. n = 8 animals for simple and 7 for complex environment.

Dendritic architecture of PrL neurons was further analyzed as a function of radial distance from soma using Sholl analysis (Fig. 4). PrL neurons from animals exposed to the complex environment exhibited an increase in the mode for the number of intersections along the dendritic profile (Fig. 4A; t_13_ = 3.81, *p* = 0.002). The mode here refers to a maximum number of intersections observed for a given neuron. Moreover, the mode for the intersection was reached nearer to the cell soma in animals with complex housing environment (Fig. 4B; t_13_ = 2.67, *p* = 0.019), suggesting a selective increase in dendritic material nearer to the cell soma. The effects of housing conditions on radial geometry of the PrL neurons were robust for both maximum intersections (Cohen’s *d* = −3.6; ∆ = 3.37 with 95% confidence intervals 1.46 to 5.28) and the radial distance where maximum intersections were observed (Cohen’s *d* = 1.37; ∆ = −8.51 with 95% confidence intervals −15.4 to −1.61).

A two-way analysis of variance was conducted with radial distance as within-subject and housing environment as a between-subject source of variance. This analysis revealed significant main effect of experimental treatments (F_(1,13)_ = 8.85, *p* = 0.011) and their interaction with the Sholl profile (F_(27,351)_ = 2.95, *p* < 0.001). Number of intersections also varied significantly with distance from the soma (F_(1,13)_ = 272.6, *p* = 0.011), which explained more than 90% of the total variance in the analysis. Dendritic intersections from 30 µm through 90 µm exhibited statistically significant inter-group differences between simple and complex housing environments (Fig. 4B; unpaired Student’s t-test). Segments nearer to the cell soma presented with greater effect sizes. Segments at 30 through 50 µm exhibited Cohen’s *d* between −3.42 to −2.35. Effects of complex housing were of a lesser magnitude at 60 through 80 µm as evidenced by lower standardized effect size (*d* = −1.92 to −1.24), which further diminished between 90 through 110 µm (*d* = −1.10 to −0.55).

### Complex environment increased dendritic spine density of prelimbic and infralimbic medial prefrontal cortex neurons

**Figure 5:**
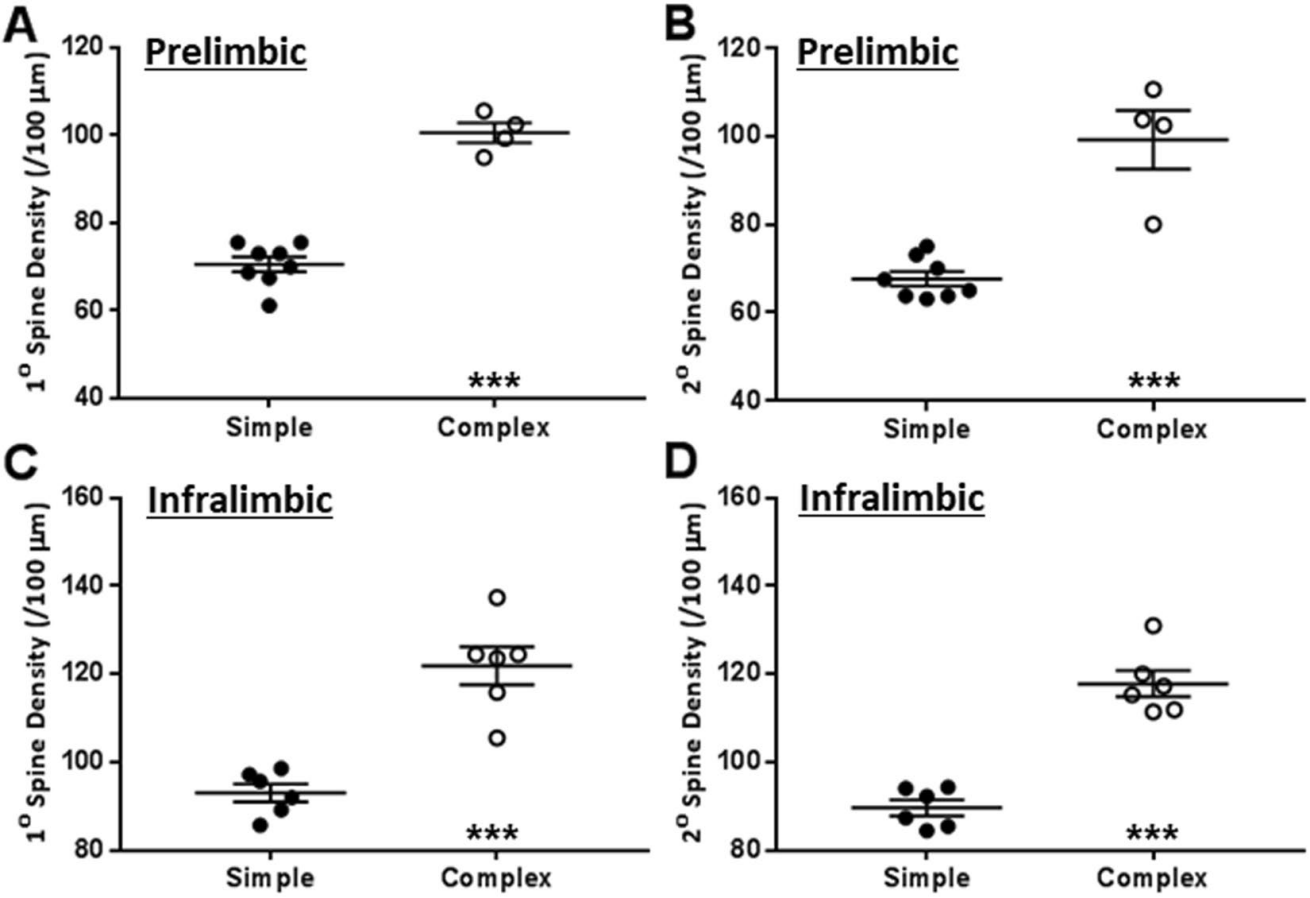
Complex environment increased density of spines of primary and secondary dendrites of the neurons in both prelimbic and infralimbic medial prefrontal cortex. ***p < 0.001; unpaired Student’s t-test. n = 8 animals for simple and 4 for complex environment (prelimbic); and, 6 animals for simple and 6 for complex environment (infralimbic).

Complex housing environment significantly increased dendritic spine density of the PrL neurons. This was congruently evident for both primary dendrites (Fig. 5A; t_10_ = 10.31, *p* < 0.001) and secondary dendrites (Fig. 5B; t_10_ = 6.26, *p* < 0.001). The effects of housing conditions on PrL spine density were substantial in the magnitude; evidenced by robust effect sizes for both primary dendrites (Cohen’s *d* = −6.39; ∆ = 24.00 with 95% confidence intervals 18.81 to 29.19) and secondary dendrites (Cohen’s *d* = −3.17; ∆ = 25.25 with 95% confidence intervals 16.26 to 34.24).

Similar to PrL, complex housing also increased dendritic spine density of IL neurons for both primary dendrites (Fig. 5C; t_10_ = 6.03, *p* < 0.001) and secondary dendrites (Fig. 5D; t_10_ = 8.15, *p* < 0.001). Similar to PrL, effects of the housing on IL spine density was of substantial effect size for both primary dendrites (Cohen’s *d* = −3.48; ∆ = 17.30 with 95% confidence intervals 10.90 to 23.69) and secondary dendrites (Cohen’s *d* = −4.71; ∆ = 16.90 with 95% confidence intervals 12.28 to 21.51).

For both dendritic segments for PrL and IL, maxima of animals from simple environment were below the minima for animals from the complex environment (Fig. 5A–D).

## Discussion

We analyzed two sub-regions within the ventromedial prefrontal cortex of the rats: namely prelimbic cortex (PrL) and infralimbic cortex (IL). Effects of complex housing or environmental enrichment on PrL or IL have not yet been studied. This is important given functional and connectional heterogeneity within rat prefrontal cortex^14^. Our choice of sub-regions in this study was guided by dichotomies between PrL and IL that are often observed during conditioned fear and drug seeking^15^. PrL is required for retrieval and expression of the conditioned fear in rats^16^, while IL is needed for retention of subsequent fear extinction^17^. Similarly, PrL is critical for reinstatement of drug-seeking^18^, while IL is involved in the extinction of cocaine seeking^19^. Observations of these dichotomies have led to a generalized notion that PrL and IL play different roles in the expression of conditioned versus extinction memories in both aversive and appetitive domains. We report here that complex environment results in facilitation of the spine density in both of these brain regions; notwithstanding their often opposing roles in the behavioral output. Our results also show that complex housing environment increases the complexity of dendritic arbors in the PrL, while its effects on IL neurons remain equivocal.

The disparate nature of effects on PrL and IL might suggest that complex housing asymmetrically and preferentially affects PrL- dependent behaviors. The evidence from reinstatement and extinction of drug-seeking does not support this suggestion. Complex housing reduces reinstatement of cocaine-seeking after a period of abstinence and increases extinction of cues associated with cocaine^20^; despite dissociable roles of PrL and IL on these behavioral components. This is congruent with increase in spine density of PrL and IL neurons in the present study. Moreover, PrL and IL are also characterized by differences in their efferent^21^. For example, PrL neurons mainly project to basolateral part of the amygdala while IL neurons project to inhibitory neurons in central amygdala and intercalated cell masses within the amygdala. Projections from amygdala to PrL and IL likewise also show regional specificity^22^. Further experiments are required to directly compare the influence of housing environment on expression and extinction of conditioned fear memories.

Effects of housing conditions reported here are of substantial magnitude. Complex environment caused a considerable increase in dendritic material, reflected in >45% increase in total dendritic length and >40% increase in spine density of PrL neurons. This translates to more than 75% more spines in PrL neurons as a result of three weeks of complex housing conditions. Moreover, the dendritic changes in the PrL are more pronounced in segments that are proximal to the cell soma, suggesting a robust effect of these changes on the electrotonic properties of the neurons.

Brain and environment are intricately interlinked. While brain participates in creating environment-relevant behaviors, the environment itself changes the brain^23^. A plethora of studies have suggested that environment can shape future behaviors by changing underlying neural substrates^24^. Such experience-dependent changes likely underlie remarkable inter-individual variability in outcomes of brain injury or age-related brain disorders^25–27^. For example, it is common to find neurodegenerative changes during the post-mortem of cognitively healthy individuals^28^, suggesting variation in the ability of neural systems to withstand challenge. In other words, variable amounts of brain and cognitive reserves can moderate effects of the aging, and perhaps other insults, on the mental functioning^29^. The concept of such reserves is often divided into brain reserves and cognitive reserves. The brain reserves pertain to structural substrates like dendritic material, number of synapses and number of neurons that can buffer the gradual attrition during the insults. Results in this report suggest that complexity of the housing conditions can increase parameters that are often taken as proxies for the brain reserve.

While enrichment effects on prefrontal architecture have not yet been studied, previous papers have investigated positive effects of complex housing (or enriched housing) on other cerebral cortices. Exposure to complex environment increases gross weight of cortex in rat brain^30^; one of the first demonstration that environment can cause macroscopic changes in the cerebral cortex. These observations were later extended by showing an increase in cortical thickness^31^ and increase in dendritic length^32^ of visual cortex. Similarly, rearing rats for three months after birth in complex housing environment cause dendritic expansion in parietal cortex pyramidal neurons^33^. These results should not be interpreted as housing causing a generalized response across the whole of the cerebral cortex. Effects of complex housing show regional specificity with disparate effects. Thus prolonged enrichment for 4–5 months did not cause any structural change in the motor cortex of mice while simultaneously enhancing the dendritic length of hippocampal neurons^34^. Similarly, twenty-one-day long exposure to a complex environment similar to current study causes dendritic retraction in basolateral amygdala^10^; a brain region that is ontologically derived from the cortical origin^35,36^. Within prefrontal cortex itself, cingulate cortex neurons do not exhibit structural dendritic plasticity in response to 3.5 months of housing in the complex environment^37^. Thus effects of complex housing on neuronal substrates appear to be idiosyncratic to individual cortical regions. Present observations show that effects of housing environment can differ even within narrow confines of the ventromedial prefrontal cortex, with disparate effects in PrL and IL. While complex housing enhanced spine density in both PrL and IL, the dendritic arbors were only increased in the PrL.

Complex housing and environmental enrichment are often interchangeably used to describe the housing akin to that described above^38^. Typical laboratory housing is characterized by a lack of opportunities to express species-typical rodent behaviors. The robust effects of complex housing environments could reflect partial recapitulation to the species-typical lived environment rather than ‘enriched’ housing per se. In this context, we chose to use the preceding description here to avoid the qualitative connotation of the enrichment. Succinct to the current observations, the medial prefrontal cortex is an important brain substrate for species-typical behavior in rodents. For example, lesions of medial prefrontal cortex reduce food hoarding in both food-deprived and *ad libitum* condition^3,39^. On the other hand, exposure to complex housing results in rapid reinstatement of species-typical behaviors in rats^40^. It remains unclear if the dendritic expansion of PrL/IL neurons is secondary to greater opportunities for expression of species-typical behavior in the complex environment.

Multiple factors can contribute to the ‘enriching’ aspect of the complex housing in the current study. These include the introduction of environmental novelty, the addition of cage-mates, greater potential of social interactions, physical activity, nutritional factors and the possibility to exhibit species-typical behaviors like territoriality or taking refuge. For example, animals in complex housing have intermittent access to sucrose in the form of fruit loops. Prolonged access to sucrose over 12 weeks can change spine density of neurons in nucleus accumbens^41^. Similarly, availability of refuge can diminish stress response in individuals during antagonistic encounters^42^, which can potentially alter dendritic features. Moreover, many of these factors can interact with each other; for example sucrose consumption and baseline stress levels. Results in this study provide a measurement of main effects of all these potential contributors and their summed interactions. A careful dissection is warranted to systematically quantify rich web of interacting factors that create the experience of the complex housing. It is also plausible that the ‘enriching’ aspect of the short paradigm used here results from emergent properties of multiple contributing factors rather than individual effects of well-defined components.

It is important to note that number of animals within a single cage in our study differed between experimental groups. While animals in simple housing lived in a dyad, animals in complex housing lived in a cohort of four animals composed of two such dyads. Our results do not dissociate if effects of complex housing were due to increase complexity of social interactions or due to other non-social components of the housing environment or the interaction between these two sources of the complexity.

There are multiple avenues by which complex housing can increase the dendritic complexity of the medial prefrontal neurons. Complex housing buffers the effect of stress. This includes plastic changes in brain structures that respond to the environmental stress, e.g., amygdala^10^. Neurons of PrL also show sensitivity to the stress^6,8^. Thus it can be proposed that either reduction in stress secondarily leads to PrL dendritic expansion, or alternatively, that dendritic expansion in PrL leads to plasticity in efferent targets like amygdala to further blunt the stress response. Similar arguments can also be made in the context of learning and memory or reward-seeking behaviors. Complex housing enhances learning and reduces responding to cues associated with rewards^20,33,37^. The complex housing also causes plastic changes in brain circuits underlying learning and drug-seeking. For example, complex housing increases neurogenesis in the hippocampus and enhances the behavioral flexibility of the spatial learning^43,44^. Plastic nature of the relation between reward system and prefrontal cortex is further buttressed by the observations that complex housing strengthens perineuronal nets in PrL and IL during abstinence in rats previously trained to self-administer sucrose^45^. Prefrontal cortex contains reciprocal connections with both hippocampus and mesolimbic dopamine system. The relative importance of these pathways and their interactions with each other can only be clarified with further sequence of experimentations. Future work is also required to determine if PrL dendritic plasticity represents a primary event that leads to efferent changes or if the expansion is secondary to changes in other brain structures related to stress regulation, learning or motivation.

Current results show that medial prefrontal cortex responds to the housing milieu of the laboratory rats. We report that exposure to complex housing results in robust neuronal hypertrophy of PrL, characterized by greater dendritic complexity and spine density compared to animals exposed to non-complex environment. These observations represent an important avenue in the search for neurological and behavioral resilience in a changing environment.

### Significance Statement

Observations in this report describe the dendritic expansion of neurons in the ventromedial prefrontal cortex in response to increase in complexity of the housing environment. This phenomenon is a structural example of the interaction between brain and living.
